# Exploring the use of bimodal transcranial direct current stimulation to enhance movement in individuals with patellofemoral pain—A sham-controlled double blinded pilot study

**DOI:** 10.3389/fnhum.2024.1427091

**Published:** 2024-09-06

**Authors:** Kai-Yu Ho, Connan Wallace, Jeno Aquino, Bryce Broadwell, Makenzie Whimple, Jing Nong Liang

**Affiliations:** Department of Physical Therapy, University of Nevada, Las Vegas, Las Vegas, NV, United States

**Keywords:** patellofemoral pain, transcranial direct current stimulation, knee valgus, non-invasive brain stimulation, cortical excitability, central activation

## Abstract

**Introduction:**

In individuals with patellofemoral pain (PFP), addressing increased knee valgus during weight-bearing activities typically involves strengthening weak hip muscles. However, recent literature highlights the role of altered descending central control in abnormal movements associated with PFP. While transcranial direct current stimulation (tDCS) has demonstrated the capacity to enhance neuroplasticity, its application targeting the corticomotor function of gluteal muscles in PFP remains unexplored. This study aimed to investigate the effects of combining bimodal tDCS with exercise on frontal plane kinematics in individuals with PFP. The hypothesis was that bimodal tDCS, specifically targeting the corticomotor function of the gluteal muscles, would augment the effectiveness of exercise interventions in improving frontal plane kinematics compared to sham stimulation.

**Methods:**

Ten participants with PFP participated in two sessions involving either bimodal tDCS or sham stimulation, concurrently with hip strengthening exercises. Weight-bearing tasks, including single leg squat, single leg landing, single leg hopping, forward step-down, and lateral step-down, were performed and recorded before and after each session. Pain visual analog scale (VAS) scores were also documented. A one-way ANOVA with repeated measures was employed to compare kinematics, while a Friedman test was used to compare VAS across the three conditions (pre-test, post-tDCS, and post-Sham).

**Results:**

We observed no significant differences in trunk lean angle, hip and knee frontal plane projection angles, or dynamic valgus index among the three conditions during the five weight-bearing tasks. VAS scores did not differ across the three conditions.

**Discussion and conclusion:**

A single session of tDCS did not demonstrate immediate efficacy in enhancing frontal plane kinematics or relieving pain in individuals with PFP. Considering observed positive outcomes in other neurological and orthopedic populations with multi-session tDCS applications, suggesting potential cumulative effects, further research is essential to explore the effects of multi-session tDCS on weight-bearing movement and underlying neurophysiology in individuals with PFP.

## 1 Introduction

Patellofemoral pain (PFP) is prevalent throughout the lifespan, affecting not only the general population but also specific populations such as adolescents, highly active individuals, and the military, with an incidence rate of 9–15% (Smith et al., 2018). Furthermore, females are 2.23 times more likely to experience PFP than males, with a prevalence of 12–13% in those ages 18–35 years (Roush and Curtis Bay, 2012). One hallmark symptom of PFP is pain around or behind the patella, which is often exacerbated by loading of the patellofemoral joint in a flexed knee position (Crossley et al., 2016; Collins et al., 2018). Individuals with PFP often exhibit difficulty performing weight-bearing tasks such as negotiating stairs, squatting, and running (Collins et al., 2018). An increase in knee valgus is a common movement deficit observed during those weight-bearing tasks in this population (Powers et al., 2017; Scholtes and Salsich, 2017). This atypical pattern is the result of excessive hip adduction and internal rotation, which causes excessive loading to the lateral aspect of patella and PFP (Powers et al., 2017; Scholtes and Salsich, 2017). As weakness of hip musculature (i.e., hip abductors and hip external rotators, specifically the gluteus medius and gluteus maximus) is believed to contribute to excessive knee valgus during weight-bearing activities (Powers et al., 2017), addressing hip muscle strength deficits via hip muscle strengthening exercises is a commonly theorized treatment for such faulty movements (Willy et al., 2019). However, while hip muscle strengthening programs have been shown to reduce pain and deficits in hip muscle weakness, the current evidence does not support the effectiveness of these programs in altering knee valgus during weight-bearing activities (Willy et al., 2019; Davis et al., 2020). This suggests that there may be structural and/or functional adaptations in the brain and/or spinal cord related to chronically persistent faulty movements in this population.

Central activation is quantified using central activation ratio, which provides a gross estimate of the number of motor units that can possibly be recruited and the extent to which these motor units can achieve maximal firing frequency (Kent-Braun and Le Blanc, 1996). Diminished central activation of hip musculature is a critical and often overlooked factor underlying impaired hip muscle performance and movement deficits in individuals with PFP (Ho et al., 2021, 2022). Our laboratory’s systematic review and meta-analysis found altered cortical reorganization in individuals with PFP, highlighting the significance of central neural control (Liang et al., 2021; Ho et al., 2022). Furthermore, reduced central activation of the gluteal muscles have been reported (Samuel, 2021), which is linked to excessive hip adduction during single-leg squat and fear-avoidance behaviors in females with PFP (Glaviano and Norte, 2021). Central activation deficits, or central inhibition, of the gluteal muscles indicate the compromised ability to activate the available muscle mass. Given that individuals with PFP may present with central activation deficits in their gluteal muscles, it is crucial for clinical interventions to target such central inhibitions (Glaviano et al., 2019).

Impaired central activation levels are associated with corticomotor deficits in patients with neuromuscular dysfunction following anterior cruciate ligament reconstruction surgeries. In individuals after anterior cruciate ligament reconstruction, those with quadriceps central activation deficits showed decreased corticomotor excitability as assessed using transcranial magnetic stimulation (TMS). On the contrary, individuals post-anterior cruciate ligament reconstruction, with central activation levels comparable to controls, exhibited equivalent corticomotor excitability (Pietrosimone et al., 2015). Consequently, enhancing corticomotor excitability is considered a potential approach to mitigate central activation failure, thereby improving weight-bearing movements in individuals with PFP (Lefaucheur et al., 2017).

Several non-invasive brain stimulation techniques and protocols have demonstrated the ability to modulate activity in particular brain regions, thereby influencing behavior, enhancing learning, improving function, and optimizing task performance. Notably, transcranial direct current stimulation (tDCS) stands out as a cost-effective and efficacious option among these methods (Coffman et al., 2012; Parasuraman and McKinley, 2014). By applying a direct weak current to the brain delivered using electrodes placed on the scalp, tDCS can modulate cortical excitability (Nitsche and Paulus, 2000; Jeffery et al., 2007), improve motor functions (Hummel et al., 2010; Dumel et al., 2016; Yi et al., 2021), or alleviate pain (Ahn et al., 2019). The neuromodulatory effect is contingent upon the placement and polarity of the electrodes on the scalp. Anodal stimulation enhances cortical excitability, cathodal stimulation reduces cortical excitability, and bihemispheric or bimodal stimulation concurrently increases excitability in the region under the anode while decreasing excitability in the region under the cathode (Nitsche and Paulus, 2000; Mordillo-Mateos et al., 2012). Moreover, previous research has indicated that bimodal tDCS over the motor cortex yields an additive effect. This augmentation is evident in facilitating motor performance in the hand contralateral to the cortex receiving anodal stimulation, surpassing the effects observed with anodal or cathodal stimulation applied in isolation (Vines et al., 2008; Klomjai et al., 2022).

While the current literature using tDCS reports promising improvements in gait, postural control and motor functions in individuals with neurological conditions, such as Parkinson’s disease, traumatic brain injuries, and stroke (Marquez et al., 2015; Lefaucheur et al., 2017; Kim et al., 2019), its application in musculoskeletal disorders remains limited. tDCS has been used to manage pain in individuals with knee osteoarthritis or those who have undergone total knee replacement, with reported improvements in functional outcomes. (Chang et al., 2017; Khedr et al., 2017; Ahn et al., 2018). In individuals with PFP, improved quadriceps muscle strength and symptom relief have been reported after a 4-week (12-sessions) anodal tDCS protocol (Rodrigues et al., 2022). Previous research examining acute effects of a single session of tDCS combined with various tasks has shown modulation of neurophysiological measures, such as corticomotor excitability and spinal circuitry, in both neurologically impaired and non-impaired cohorts (Nitsche and Paulus, 2000; Bastani and Jaberzadeh, 2012; Fernández-Lago et al., 2017). Furthermore, bimodal tDCS has demonstrated improved lower limb function after a single session in post-stroke individuals during the subacute phase of recovery (Tahtis et al., 2014). Given the unclear efficacy of a gluteal central activation paradigm employing tDCS to enhance gluteal activation, hip kinematics, hip muscle strength, function, and pain in individuals with PFP, we aim to explore the effects of a single session of bimodal tDCS in this population.

With respect to the effects of pain relief, non-invasive brain stimulation studies, aiming to modulate pain through neuromodulation effects, have often targeted the primary motor cortex (M1) contralateral to the site of pain. Multiple studies have reported pain reduction following various repetitive TMS protocols of M1 in various types of clinical pain (Lefaucheur et al., 2004; Passard et al., 2007; Goto et al., 2008; Hosomi et al., 2013), as well as alter pain thresholds and cortical representations (Houzé et al., 2013). Moreover, neuroimaging studies have demonstrated that the changes induced by motor cortex stimulation are not confined to the motor system alone. Instead, these changes extend to cortical and subcortical areas involved in pain processing and modulation (García-Larrea et al., 1999; Bestmann et al., 2004; Peyron et al., 2007).

Therefore, this study aimed to explore whether single session of bimodal tDCS over the motor cortices paired with exercise could alleviate PFP and enhance frontal plane kinematics in the lower extremity and trunk during weight-bearing tasks for individuals with PFP. Our hypothesis was that the combination of bimodal tDCS targeting the corticomotor area of the gluteal musculature with exercise would be more effective in alleviating pain and improving frontal plane kinematics compared to exercise with sham stimulation.

## 2 Materials and methods

### 2.1 Recruitment and participants

We enrolled 10 individuals with PFP, characterized by peri- and/or retro-patellar pain persisting for a minimum of 3 months (Crossley et al., 2016). With a Type I error of 0.05, a power of 95%, and a calculated effect size of 0.82, this sample size was deemed sufficient for detecting a reduction in knee valgus following an acute intervention protocol (Scholtes and Salsich, 2017). Participants were recruited through advertisement flyers and email outreach in the Las Vegas area from 2022 to 2023. Prior to participation, all participants were informed about the study’s procedures and signed an informed consent form approved by the Institutional Review Board of the University of Nevada, Las Vegas. Clinical trial registration: https://clinicaltrials.gov/, NCT06565520.

Inclusion criteria encompassed a knee valgus presentation (assessed via a forward step-down test) (Earl et al., 2007; Park et al., 2013; Lopes Ferreira et al., 2019; Ho and Murata, 2021), predominantly unilateral PFP for at least 3 months, and an age range of 18–45 years. Although our primary focus was on individuals with unilateral PFP, we also included participants reporting PFP on both limbs if they consistently experienced greater pain duration and magnitude unilaterally over the past 3 months. This decision aligned with our tDCS protocol, aiming to enhance the cortical representation of gluteal muscles on the affected limb while inhibiting the contralateral limb.

Furthermore, participants underwent a physical examination to rule out concomitant sources of pain. This process involved palpation of the soft tissues around the patellofemoral joint and a patellar compression test (Nijs et al., 2006) to identify the location of pain. The patellar compression test entailed pressing the patella distally when the participant straightened their knee (Nijs et al., 2006), and participants were excluded if the knee pain did not originate from their patellofemoral joint. Physical examinations were performed by a Doctor of Physical Therapy student who was trained by a licensed physical therapist and musculoskeletal researcher.

Exclusion criteria included a history of any traumatic patellar dislocation or knee surgery (Ho et al., 2021). Additionally, a safety screening questionnaire was administered for tDCS safety prior to recruitment (Charalambous et al., 2019; Liang et al., 2020). The safety and ethical guidelines for TMS in clinical practice and research are encapsulated in a 13-item questionnaire developed to screen candidates prior to TMS application. To ensure maximum safety, we utilized this questionnaire to screen participants before applying tDCS. The questionnaire includes inquiries about the history of epilepsy or seizures, head trauma, loss of consciousness, implants, medications, and any past issues experienced during TMS or magnetic resonance imaging procedures (Rossi et al., 2011).

### 2.2 Procedures

This study employed a double-blinded, sham-controlled crossover design. Each participant attended two sessions separated by 14 days. The stimulation type (bimodal tDCS or sham) for the first participant was determined by a coin flip. Subsequently, each participant alternated the stimulation type for their first session. For example, if the first participant received sham stimulation first, the second participant received bimodal stimulation first, and the third participant received sham stimulation first. This process continued such that five participants received bimodal tDCS in their first session, and the remaining five received sham stimulation in their first session. During their second session, each participant was given the opposite stimulation of what they were given during their first session.

Before the initial stimulation session, participants completed the Global Physical Activity Questionnaire (GPAQ) to determine their physical activity level (Bull et al., 2009) and the Anterior Knee Pain Scale (AKPS) to assess functional activity (Watson et al., 2005). The AKPS, a reliable self-report tool, measures function in individuals with PFP through 13 weighted questions, with a score of 100 indicating no disability (Watson et al., 2005). Additionally, participants’ height and mass were recorded.

### 2.3 tDCS application and exercise protocol

**FIGURE 1:**
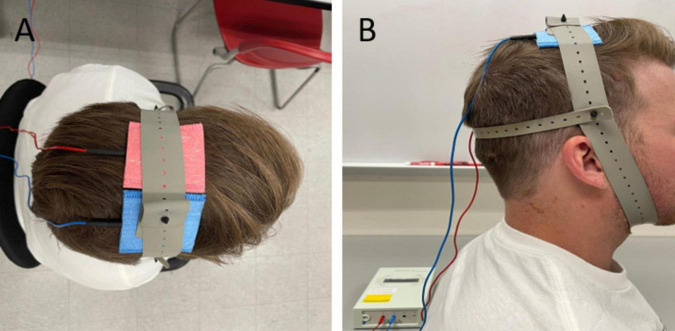
Bihemispheric tDCS montage or bimodal tDCS in the superior **(A)** and lateral **(B)** view. Anode (red) positioned over the primary motor cortex contralateral to the affected limb and cathode (blue) positioned above the ipsilateral cortex.

Throughout the study, a single researcher was responsible for administering the tDCS or sham stimulations to all participants. This researcher exclusively knew the type of stimulation each participant received in each session. The other researchers, blinded to the stimulation types, conducted assessments for outcome measures. Participants remained blinded to the type of stimulation administered during each session. A direct current stimulator (NeuroConn DC-Stimulator PLUS, Germany) was used to deliver a weak direct current via two conductive electrodes placed in saline soaked sponges (5 cm x 5 cm). tDCS was administered with the anode over the primary motor cortex contralateral to the affected limb and the cathode over the ipsilateral motor cortex. The medial border of each electrode was positioned 5 mm lateral to the Cz, following the international 10–20 electroencephalography (EEG) system conventions (Tahtis et al., 2014). Care was taken to ensure that the two electrodes did not come into contact at any point during the experimental session (Figure 1). The tDCS application involved a 30-s ramp-up using a 2-mA current, 19 min of stimulation, and a 30-s ramp-down. For sham stimulation, a similar arrangement was used, but the 2mA current was ramped up for 30 sec, after which the current was gradually ramped down and turned off for the remaining time (Waters-Metenier et al., 2014; Liang et al., 2020). This brief stimulation period mimics the cutaneous perception of the tDCS stimulation, and thus blinds the participants to the type of stimulation they received but does not change the cortical excitability (Gandiga et al., 2006; Nitsche et al., 2008).

**FIGURE 2:**
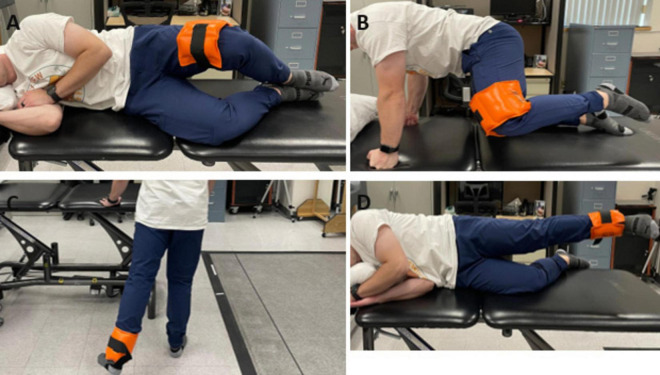
Hip strengthening exercises employed during tDCS/sham stimulation: **(A)** clamshells **(B)** quadruped hip abduction **(C)** standing 45° hip extension **(D)** side-lying hip abduction.

During the tDCS stimulation period, participants completed 3 sets of 12 repetitions for exercises including clamshells, quadruped hip abduction, standing 45° hip extension, and side-lying hip abduction (Figure 2). Rest periods of 30 s between sets and 1 min between exercises were given. Exercise resistance was applied using an ankle weight set at 30% of their one-repetition maximum (1RM), determined through a handheld dynamometer (Kraemer et al., 2002). This resistance remained consistent across conditions, established during the initial session. Such prescriptions were established based on the American College of Sports Medicine guidelines for resistance training in healthy adults (Kraemer et al., 2002), which recommend using light loads (30–60% of 1 RM) performed for multiple sets per exercise. We chose 30% of 1 RM based on pilot testing with two participants with PFP. They reported that 40% or higher resistance was too challenging to complete the required repetitions and sets.

### 2.4 Weight-bearing tasks

**FIGURE 3:**
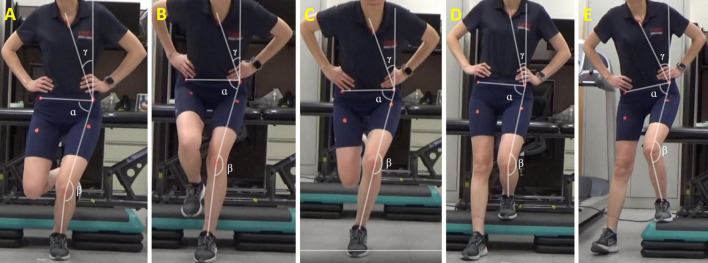
Two-dimensional frontal plane kinematics measured during **(A)** single leg squat, **(B)** single leg landing, **(C)** single leg hopping, **(D)** forward step-down, and **(E)** lateral step-down tasks. TLA = ρ; hip FPPA = α; knee FPPA = β; DVI = α + β. FPPA, frontal plane projection angle; DVI, dynamic valgus index; TLA, trunk lean angle.

Before and immediately after the tDCS/sham stimulation paired with exercises, we captured frontal plane videos of participants performing five weight-bearing tests. Participants were given a 5-min break between the exercise session and the weight-bearing task session to avoid fatigue. Although additional rest time was allowed if needed, none of the participants requested it. Additionally, participants’ pain levels before and after each session were quantified using the Visual Analog Scale (VAS), with zero denoting no pain and 10 indicating maximum pain. The VAS has demonstrated reliability, validity, and responsiveness in assessing symptoms in individuals with PFP (Cowan et al., 2002). The five tasks included the single-leg squat, single-leg landing, single-leg hopping, forward step-down, and lateral step-down (Figure 3). Using a SonyCX405 Handycam^®^ positioned 15 feet anteriorly from the participant, we captured frontal plane trunk and lower extremity kinematics during these weight-bearing tasks. Markers were placed over the sternum, bilateral anterior superior iliac spine (ASIS), ipsilateral patella, and bisection of ipsilateral malleoli to facilitate precise kinematic measurements.

For the single-leg squat test, participants stood on their symptomatic limb, squatted to 45° of knee flexion, and returned to the starting position over a 3-s period (Rees et al., 2019). The single-leg landing task involved participants standing on a 30-cm step with their symptomatic limb, hopping off onto a mark 30-cm forward from the step, and maintaining balance for 3 s after landing (Ho et al., 2019). In the single-leg hopping task, participants stood on their symptomatic limb, hopped as far forward as possible, and maintained balance for 3 s after landing (Ho et al., 2019). The forward step-down test required participants to stand on a 30-cm step with their symptomatic limb, lightly tap the heel of the contralateral foot on the floor in an anterior direction while maintaining balance on the platform, and return to the starting position over a 3-s period (Lee and Powers, 2014). Similarly, for the lateral step-down test, participants stood on a 30-cm step with their symptomatic limb, lightly tapped the heel of the contralateral foot on the floor in a lateral direction while maintaining balance on the platform, and returned to the starting position over a 3-s period. Each task was performed three times, with a 1-min break between tasks. During these tasks, two trained Doctor of Physical Therapy students stood on either side in close proximity as spotters, ensuring safety in case of a loss of balance. Participants were granted additional breaks upon request to prevent fatigue.

### 2.5 Data analysis

Frontal plane kinematics during the five weight-bearing tasks were assessed using Kinovea software by the same investigator, who was blinded to the condition. Four key measures were derived from video recordings, including trunk lean angle (TLA), knee frontal plane projection angle (FPPA), hip FPPA, and dynamic valgus index (DVI). The angle measurements were taken at the peak of knee flexion (Scholtes and Salsich, 2017). Initiating the measurement process, a vertical reference line was drawn superiorly from the ipsilateral ASIS. To establish the pelvic segment, a line connecting markers on bilateral ASIS landmarks was drawn. The thigh segment was delineated by bisecting the thigh with a line from the midpoint of the patella to the ipsilateral ASIS. The shank segment was formed by drawing a line from the midpoint of the patella to the midpoint of the ankle.

TLA, representing the angle between the vertical reference line and the line connecting the ipsilateral ASIS and the sternal marker, was calculated (Dingenen et al., 2014). A lower TLA indicates greater ipsilateral trunk lean toward the symptomatic limb. TLA has demonstrated agreement and reliability in comparison to a 3D motion capture system for single-leg movements (Dingenen et al., 2014). Knee FPPA was calculated by subtracting the angle between the thigh and shank segments from 180°. A higher knee FPPA indicates greater knee valgus of the symptomatic limb (Scholtes and Salsich, 2017). Hip FPPA was calculated by subtracting the angle between the pelvic and thigh segments from 90°. A higher hip FPPA indicates greater hip adduction of the symptomatic limb (Scholtes and Salsich, 2017). Considering the concurrent presence of knee valgus and hip adduction in individuals with PFP during weight-bearing tasks, the DVI was computed as the sum of the knee FPPA and hip FPPA (Scholtes and Salsich, 2017; Figure 3). A higher DVI angle indicates a greater summation of knee valgus and hip adduction. DVI exhibits a stronger correlation with kinematics measured by a 3D motion capture system compared to knee FPPA alone (Scholtes and Salsich, 2017). We measured the four angles from each of the three repetitions for every task, and the statistical analysis was conducted on the average value for each task.

### 2.6 Statistical analysis

The outcome measures of this study included hip FPPA, knee FPPA, DVI, and TLA during five weight-bearing tasks, as well as VAS before and after tDCS or sham intervention. To establish a baseline for the participants’ pre-intervention data, we calculated the average of the data collected on two separate days. This approach was employed after confirming that there was no statistically significant difference between the pre-intervention data from the two days, as determined by a paired *t*-test.

We conducted a Shapiro-Wilk test to assess the distribution of the collected data. The results indicated that the hip FPPA, knee FPPA, DVI, and TLA data followed a normal distribution. However, the VAS data exhibited a non-normal distribution. Therefore, one-way ANOVAs with repeated measures and *post-hoc* pairwise comparisons with Bonferroni corrections were used to compare the hip FPPA, knee FPPA, DVI, and TLA among the three conditions (pre-intervention, post-tDCS, and post-Sham). Friedman test was used to compare the VAS among pre-, post-tDCS, and post-Sham conditions. All statistical analyses were performed using SPSS software (ver. 27, International Business Machines Corp. New York, USA). A significant difference was defined as *p* < 0.05.

## 3 Results

### 3.1 Participant demographics

We included 6 males and 4 females with PFP (mean age = 28.2 ± 6.88 years old). Their average AKP score was 79.1 ± 7.29. The average body mass index (BMI) among participants was 26.6 ± 6.8 kg/m^2^ with an activity level of 2017.0 ± 1740.3 MET.min/week as measured by the GPAQ. Among these individuals, 8 had unilateral pain and 2 had bilateral pain. The individuals with bilateral PFP reported a history of consistently greater magnitude of pain perceived on one limb for the past 6 months.

### 3.2 Frontal plane kinematics

For single leg squat, single leg hopping, forward step-down, and lateral step-down tasks, the one-way ANOVA with repeated measures revealed no significant difference in hip FPPA, knee FPPA, DVI, and TLA among the pre-, post-tDCS, and post-Sham conditions (*p* > 0.05) (Table 1).

**TABLE 1:** Comparisons of frontal plane kinematics between pre-, post-tDCS, and post-Sham conditions during the five weight-bearing tasks.

	Pre (mean ± SD)	Post-tDCS (mean ± SD)	Post-Sham (mean ± SD)	*p*
**Single Leg Squat**				
Hip FPPA	9.63 ± 3.21°	9.86° ± 3.09°	11.84° ± 4.38°	0.38
Knee FPPA	1.92 ± 6.34°	1.06° ± 4.37°	2.06° ± 6.95°	0.76
DVI	11.22 ± 8.84°	10.92° ± 6.76°	13.90° ± 12.43°	0.44
TLA	12.19 ± 3.88°	12.69° ± 3.73°	13.47° ± 6.46°	0.55
**Single Leg Landing**				
Hip FPPA	8.81° ± 6.82°	8.96° ± 7.86°	7.92° ± 8.87°	0.74
Knee FPPA	7.73° ± 5.95°	5.72° ± 7.45°	3.70° ± 6.18°†	0.01*
DVI	16.54° ± 12.12°	14.68° ± 14.77°	11.61° ± 14.12°	0.15
TLA	12.14° ± 7.92°	11.43° ± 6.74°	10.50° ± 8.61°	0.28
**Single Leg Hopping**				
Hip FPPA	7.76° ± 6.41°	7.5° ± 7.46°	9.07° ± 9.09°	0.63
Knee FPPA	3.55° ± 4.67°	2.07° ± 6.77°	3.72° ± 7.64°	0.66
DVI	11.44° ± 12.21°	9.57° ± 13.47°	12.79° ± 15.30°	0.53
TLA	10.22° ± 6.54°	9.44° ± 7.40°	11.34° ± 10.85°	0.70
**Forward Step-Down**				
Hip FPPA	18.44° ± 4.99°	19.1° ± 5.21°	19.03° ± 6.89°	0.90
Knee FPPA	0.87° ± 10.54°	–0.05° ± 11.83°	–0.51° ± 12.87°	0.62
DVI	19.32° ± 13.12°	19.05° ± 15.42°	18.51° ± 18.6°	0.95
TLA	11.39° ± 5.07°	11.74° ± 5.01°	12.94° ± 6.41°	0.16
**Lateral Step-Down**				
Hip FPPA	25.71° ± 4.66°	24.64° ± 6.35°	26.24° ± 6.63°	0.40
Knee FPPA	17.10° ± 11.24°	14.8° ± 11.09°	16.44° ± 15.48°	0.43
DVI	42.81° ± 14.34°	39.44° ± 15.42°	42.68° ± 20.57°	0.36
TLA	15.43° ± 5.43°	13.01° ± 6.44°	14.82° ± 6.99°	0.09

*Highlights a significant difference using one-way ANOVA with repeated measures.

^†^Highlights a significant difference from the pre-condition. SD, standard deviation; FPPA, frontal plane projection angle; DVI, dynamic valgus index; TLA, trunk lean angle.

For the single leg landing task, a significant difference in knee FPPA was observed between the three conditions (pre, post-tDCS, and post-Sham) (*p* = 0.011). Post-hoc pairwise comparisons indicated a significantly lower knee FPPA after sham intervention compared to the pre-condition (*p* = 0.018). No significant difference was found between the pre-condition and post-tDCS condition (*p* = 0.394) or between post-tDCS and post-Sham conditions (*p* = 0.365) in knee FPPA (Table 1). Additionally, one-way ANOVA with repeated measures demonstrated no significant difference in hip FPPA, DVI, and TLA during single leg landing among the pre-, post-tDCS, and post-Sham conditions (*p* > 0.05) (Table 1).

### 3.3 Pain

The Friedman test showed that there was not a significant difference in VAS among the pre-intervention, post-tDCS, and post-Sham conditions (pre-intervention = 1.78; post-tDCS = 2.44; post-Sham = 1.78; *p* = 0.147).

## 4 Discussion

To our knowledge, this study is the first to investigate the immediate effects of bimodal tDCS targeting gluteal corticomotor function on frontal plane kinematics during weight-bearing tasks and pain in individuals with PFP. We hypothesized that tDCS targeting the corticomotor area of the gluteal musculature, paired with exercise, would yield better results in improving frontal plane kinematics compared to exercise with sham stimulation. Our findings showed that a single 20-min bout of tDCS did not improve frontal plane movements or pain in persons with PFP when compared to their pre-intervention state, which did not support our hypothesis.

In previous research investigating the immediate effects following a single session of tDCS application combined with various tasks, efficacy has been demonstrated in modulating neurophysiological measures. This includes corticomotor excitability (Nitsche and Paulus, 2000; Bastani and Jaberzadeh, 2012), and spinal circuit functions (Fernández-Lago et al., 2017), across diverse populations. For instance, individuals with Parkinson’s disease exhibited improved reciprocal Ia inhibition reflex following an acute session of treadmill walking combined with anodal tDCS applied to the leg motor cortex (Fernández-Lago et al., 2017). Furthermore, anodal tDCS has shown to enhance corticomotor excitability in both healthy individuals and those post-stroke (Bastani and Jaberzadeh, 2012). While the immediate impacts showcase the potential of tDCS in influencing neurophysiological parameters, the improvements in motor function, as well as retention effects, seem to be more consistently realized through the implementation of repeated sessions over an extended timeframe (Duarte Nde et al., 2014; Allman et al., 2016; Dumel et al., 2016; Goodwill et al., 2016; Alisar et al., 2020). This observation underscores the importance of considering the temporal aspects and cumulative effects of tDCS applications when evaluating its potential for enhancing motor function. Particularly in the PFP population, where chronic adaptations occur in the central nervous system in response to chronic pain and altered movement patterns peripherally, extended interventions may be required to reverse these maladaptations, in distinct contrast with populations such as stroke and Parkinson’s disease, where adaptations in the central nervous system result from lesions and degeneration in the brain.

Specific to populations with PFP, Rodrigues et al. (2022) have presented findings on the efficacy of tDCS targeting the quadriceps in combination with exercise programs, comparing it to sham stimulation with exercise programs. Their protocol included 12 sessions of anodal tDCS over 4 weeks, specifically targeting the quadriceps motor cortex. In each session, participants participated in 3 sets of 12 repetitions of knee extension exercises, with resistance set at 60% of their 10 RM. In contrast to our study findings, Rodrigues et al. (2022) reported not only improvements in knee extensor strength but also a reduction in pain after 12 sessions of tDCS intervention. This discrepancy could be due to the difference in the tDCS protocols, as our study utilized a single session, while the positive outcomes in pain reduction were observed in the context of a multi-session tDCS program. Another possible factor contributing to the different effects of tDCS is that Rodrigues et al. (2022) utilized a different level of resistance (60% of 10 RM) compared to our resistance (30% of 1 RM). While it is unclear which loading is more optimal, exploring different levels of resistance should be done in future work.

Our results revealed a significant decrease in knee FPPA during the single leg landing task post-sham intervention compared to the pre-intervention condition. Although this improvement could be attributed to the exercise, it might lack clinical significance or meaningful translation to enhanced weight-bearing task performance. Specifically, the observed 4° difference falls below the standard error of measurement (SEM) of 4.34° and the minimum detectable change (MDC) of 12.03°, both calculated using pre-intervention measurements from two sessions (pre-tDCS and pre-Sham). These statistically significant findings might also be influenced by false positives resulting from multiple comparisons (Ranganathan et al., 2016).

Compared to the larger dataset reported by Alrayani et al. (37 PFP and 278 controls) (Alrayani et al., 2023), our study demonstrated better kinematics in our participants with PFP during single-leg squat. Specifically, our results showed a hip FPPA of 9.63° and a knee FPPA of 1.92°, whereas Alrayani’s study reported a hip FPPA of 11.16° and a knee FPPA of 7.36°. This difference may be attributed to the lack of an established threshold for altered kinematics in our participant recruitment process. Additionally, the kinematics observed in our smaller sample size may not accurately represent the typical kinematics of this population. Future larger-scale studies should aim to define specific thresholds to better identify PFP participants with movement deficits, as this subgroup with faulty movement patterns may be more responsive to our tDCS protocol.

While the efficacy of a single session of various tDCS montages in modulating neurophysiological measures, such as corticospinal excitability, has shown promise in a diverse population, their impact on central activation ratio remains less comprehensively understood. Limited literature in this domain includes a study focused on post-ACL reconstruction, wherein a single session of tDCS, although lacking clarity on stimulation intensity or montage specifics, proved ineffective in enhancing quadriceps muscle function (Rush et al., 2020). This emphasizes the need for future investigations to systematically explore the effects of different tDCS montages on impaired gluteal muscle central activation within the PFP population. Additionally, it is crucial to discern between single-session and multi-session effects and elucidate how these parameters correlate with improvements in overall function. By addressing these aspects, we can enhance our understanding of the impact of tDCS on neurophysiological and functional outcomes in populations with PFP, paving the way for more targeted and effective interventions.

We acknowledge several limitations in our study. Firstly, we assessed only frontal plane kinematics through video analysis, potentially overlooking alterations in other planes, such as trunk flexion in the sagittal plane, in response to tDCS. Additionally, the lack of significant kinematic differences observed in our participants and the small sample size in this study are important considerations. Secondly, our use of EEG coordinates for electrode placement does not account for individual topographic variability across different individuals and control/patient cohorts. Future studies could utilize TMS to locate the motor hotspots for the gluteal muscles in each individual, thereby enabling more specific and targeted electrode placement. Moreover, employing TMS to quantify underlying changes in corticospinal excitability before and after tDCS can ensure effectiveness of neuromodulation.

## 5 Conclusion

This is the first study to examine the effects of tDCS targeting gluteal muscles affect frontal plane kinematics and pain in persons with PFP. Our study found that a single session of bimodal tDCS was ineffective at improving frontal plane movements or pain during weight-bearing tasks in individuals with PFP. While we did observe a reduction in knee FPPA following single session of exercise and sham stimulation, the difference was likely not clinically meaningful. Nevertheless, our work provided some preliminary insights into the acute effects of bimodal tDCS on individuals with PFP. Future research is needed to understand the effects of multi-session tDCS, varying levels of exercise resistance, and different tDCS montages on kinematics, pain, and function in persons with PFP who exhibit more pronounced faulty frontal plane movements.

## Data Availability

The original contributions presented in the study are included in the article/supplementary material, further inquiries can be directed to the corresponding author.
